# Estimating Carbon Dioxide Emissions and Direct Power Consumption of Linear Accelerator–Based External Beam Radiation Therapy

**DOI:** 10.1016/j.adro.2022.101170

**Published:** 2022-12-31

**Authors:** Rachel F. Shenker, Timothy L. Johnson, Marcio Ribeiro, Anna Rodrigues, Junzo Chino

**Affiliations:** a Department of Radiation Oncology, Duke University School of Medicine, Durham, North Carolina; b Nicholas School of the Environment, Duke University, Durham, North Carolina; c Institute of Energy and Environment, University of São Paulo, São Paulo, Brazil

## Abstract

**Purpose:**

Climate change is one of the direst health threats that humanity faces. We aim to estimate the carbon dioxide (CO_2_) emissions associated with the energy usage from linear accelerator (LINAC)-based external beam radiation therapy (EBRT) for the most common cancer diagnoses.

**Methods and Materials:**

We identified patients with the 4 most common cancer types treated with curative-intent EBRT. Beam-on time for each fraction was extracted from the treatment planning system and averaged over each site and treatment modality. The power was multiplied by the beam-on time in hours to yield kilowatt hours (kWh). Using the US Environmental Protection Agency Greenhouse Gas Equivalencies calculator, we converted the kWh into estimates of CO_2_-equivalent emissions for the average US power grid. Idle time of the LINAC was estimated via Varian Medical Systems.

**Results:**

A total of 10 patients were included for each of the following modalities: conventionally fractionated for prostate cancer (28 fractions [fx]), prostate stereotactic body radiation therapy (SBRT) (5 fx), 15- and 5-fx regimens for early-stage breast cancer, 3- and 5-fx SBRT regimens for early-stage lung cancer, conventional EBRT (30 fx) for locally advanced lung cancer, and short- (5 fx) and long-course (25-28 fx) for rectal cancer. The modality with the lowest and highest carbon emissions per course, on average, was prostate SBRT (2.18 kg CO_2_; interquartile range, 1.92-2.30) and conventional treatment for prostate cancer (17.34 kg CO_2_; interquartile range, 10.26-23.79), respectively. This corresponds to CO_2_-equivalent emissions of driving an average of 5.4 miles and 41.2 miles in a standard vehicle, respectively. “Standby” mode for a LINAC TrueBeam and Clinac IX uses 112 kWh and 64.8 kWh per day, respectively.

**Conclusions:**

We have estimated CO_2_ emissions arising from direct energy usage of a LINAC for 4 common cancers treated with EBRT. “Standby” mode of a LINAC uses the most energy per day. Comprehensive studies are warranted to minimize the environmental effects of health and cancer care.

## Introduction

Climate change is one of the direst health threats that humanity faces.^1^ The health care sector in the United States is responsible for 8.5% of greenhouse gas (GHG) emissions of the nation.^2^ In response, there is a call to action to provide more sustainable oncology practices and reduce the carbon footprint of health care.^3^ Because approximately 50% of patients who receive a diagnosis of cancer receive some form of radiation therapy (RT) throughout their illness,^4^ there is great interest in understanding the potential environmental effect of RT.

To date, there have been no published studies on the carbon dioxide (CO_2_) emissions of RT on a patient-specific level in the United States. Within this study, we aim to report the energy use and associated CO_2_ emissions of a linear accelerator (LINAC) during the delivery of external beam radiation therapy (EBRT) for 4 common cancer diagnoses. Although not a measure of the total carbon footprint of RT, energy use accounts for a significant share of related CO_2_ emissions, and accounting for it is an important first step in understanding the broader environmental effects of cancer treatment.

## Methods and Materials

This study was approved by our institutional review board of Duke University. We identified patients who completed treatment from January 2021 to June 2022 in the 4 most common cancer types treated with curative-intent EBRT (ie, prostate, breast, lung, and rectal cancer). We identified 10 patients for each group who were treated with EBRT as clinically appropriate. From the start of our study, patients were chosen if the entire prescribed course was completed and deemed to have typical treatment volumes by a clinician, until 10 were collected. Beam-on time for each fraction was extracted from the treatment planning system and averaged over each site and treatment modality. Patients were treated on either a Clinac IX or TrueBeam (Varian Medical Systems, Palo Alto, CA) LINAC (true power of 45 kW and 48 kW, respectively). This information was provided by Varian Medical Systems and verified with the departmental clinical engineer. The power was multiplied by the beam-on time in hours to yield kilowatt hours (kWh). For the purposes of this analysis, we assumed that the power grid supplies all electricity consumed. In reality, a small fraction of our estimate is “reactive” power supplied locally (ie, our power estimate is actually “apparent power,” and the power factor is less than 1.0). This assumption yields a conservative upper bound on electricity consumption and associated CO_2_ emissions.

Idle time of the TrueBeam LINAC, including “Standby” (7 kW), “On–No Mode” (11 kW), and “Ready” (15 kW) positions was estimated using information provided by Varian Medical Systems and a typical schedule of patients treated on a machine in our department. Similarly, idle time of the Clinac IX LINAC for “Standby” and “Ready” was reported to be 3 kW and 20 kW, respectively. Based on a typical treatment-day schedule and estimates provided by Varian, a TrueBeam LINAC was assumed to be in “Ready” state for 0.8 hours per day, “On–No mode” at 5.6 hours per day, and “Standby” at 16 hours per day. For the Clinac IX, a LINAC was assumed to be in “Ready” state for 0.8 hours per day and “Standby” for 21.6 hours per day.

Using the US Environmental Protection Agency (EPA) Greenhouse Gas Equivalencies calculator, the kWh was converted into estimates of CO_2_ equivalent emissions using a national average electric power emissions intensity of 0.238 kg CO_2_/kWh.^5^ To remain within the scope of our study, the energy use of any diagnostic or treatment verification imaging was not included.

## Results

A total of 10 patients were included for each of the following modalities: conventionally fractionated for prostate cancer (28 fractions [fx]), prostate stereotactic body radiation therapy (SBRT) (5 fx), 15- and 5-fx regimens for early-stage breast cancer, 3- and 5-fx SBRT regimens for early-stage lung cancer, conventional EBRT (30 fx) for locally advanced lung cancer, and short- (5 fx) and long-course (25-28 fx) for rectal cancer (total n = 90).

**Figure 1:**
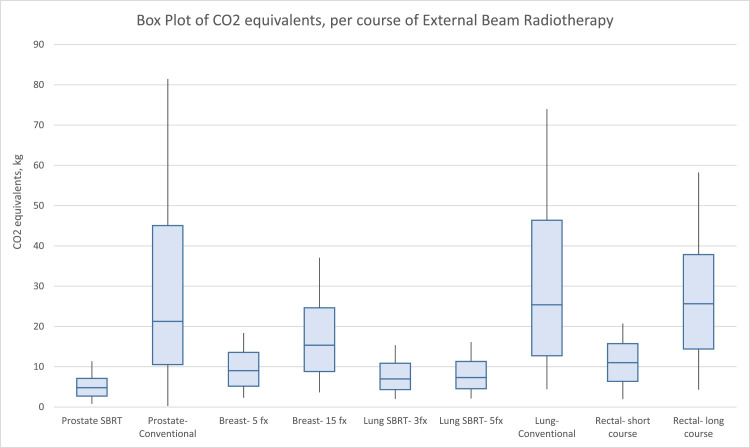
Box plot of CO_2_ equivalents, per course of external beam radiation therapy. *Abbreviations:* CO_2_ = carbon dioxide; SBRT = stereotactic body radiation therapy.

The modality with the lowest and highest carbon emissions per course, on average, is prostate SBRT (2.18 kg CO_2_; interquartile range [IQR], 1.92-2.30) and conventional treatment for prostate cancer (17.34 kg CO_2_; IQR, 10.26-23.79), respectively. These values correspond to CO_2_-equivalent emissions of driving on average 5.4 miles and 41.2 miles in a standard vehicle, respectively. After prostate SBRT, the next 3 modalities with the lowest carbon emissions on average were 3-fx lung SBRT (3.0 kg CO_2_; IQR, 2.33-3.88), 5-fx lung SBRT (3 kg CO_2_; IQR, 2.43-4.00), and 5-fx breast (3 kg CO_2_; IQR, 2.86-4.51). After conventional treatment for prostate cancer, the next 3 modalities with the greatest carbon emissions on average were conventional EBRT for lung cancer (14.42 kg CO_2_; IQR, 8.32-20.99), long-course rectal cancer (11.32 kg CO_2_; IQR,10.11-12.21), and 15-fx breast cancer (7.19 kg CO_2_; IQR,5.18-9.30). These results are summarized in Table 1 and represented in Fig. 1.

**Table 1:** Fraction and course time, greenhouse gas, and CO_2_ emissions of the 4 most common cancers by modality from lowest to highest by course time

Site	Modality	Number of fractions	Fraction time, min, average (range)	Whole course time, min (range)	kWh per course, average (range)	GHG-equivalent emissions, miles driven, average (range)	Carbon equivalents per course, CO_2_ kg, average (range)
Prostate	SBRT	5	1.26 (0.46–2.47)	6.29 (2.29–12.34)	5.03 (1.83–9.88)	5.4 (2–10.6)	2.18 (0.79–4.27)
Lung	SBRT	3	2.88 (1.91–4.21)	8.65 (5.73–12.63)	6.92 (4.58–10.1)	7.4 (4.9–10.8)	3.0 (1.98–4.50)
Lung	SBRT	5	1.83 (1.22–2.79)	9.15 (6.10–13.93)	7.32 (4.88–11.15)	7.9 (5.2–12)	3.17 (2.11–4.82)
Breast	Hypofractionated	5	2.11 (1.34–2.79)	10.56 (6.68–13.95)	8.45 (5.34–11.16)	9.1 (5.7–12)	3.65 (2.34–4.83)
Rectal	Short course	5	2.52 (1.12–2.89)	12.6 (5.61–14.47)	10.08 (4.49–11.57)	10.8 (4.8–12.4)	4.36 (1.95–5.00)
Breast	Hypofractionated	15	1.39 (0.70–2.39)	20.79 (10.51–35.89)	16.63 (8.41–22.56)	17.9 (9–24.2)	7.19 (3.63–12.42)
Rectal	Long course	25-28	1.23 (0.46–2.18)	32.7 (12.43–58.96)	26.16 (9.94–47.17)	28.1 (10.7–50.7)	11.32 (4.29–20.41)
Lung	Conventional	30	1.41 (0.42–2.83)	42.18 (12.73–85.02)	33.32 (10.18–63.77)	35.8 (10.9–68.5)	14.42 (4.41–27.63)
Prostate	Conventional	28	1.88 (0.83–3.22)	52.68 (23.25–114.24)	38.34 (18.6–85.68)	41.2 (20–92)	17.34 (0.28–`36.4)

*Abbreviations:* CO_2_ = carbon dioxide; SBRT = stereotactic body radiation therapy.

The estimated idle time of the LINAC also was reported for a working day. For the TrueBeam, the power used for LINAC “Ready,” “On–No mode,” and “Standby” was estimated to be 12 kWh, 62 kWh, and 112 kWh per day. This equates to emissions of 5.2 kg CO_2_, 26.8 kg CO_2_, and 48.5 kg CO_2_, respectively. On a nonwork day, the LINAC was assumed to be in “Standby” for 24 hours for 2 days, yielding 168 kWh for a weekend, equating to emissions of 72.7 kg CO_2_. For the Clinac IX, the power used for LINAC “Ready” and “Standby” was estimated to be 16 kWh and 64.8 kWh, equating to emissions of 6.9 kg CO_2_ and 28 kg CO_2_, respectively.

## Discussion

This study reports the estimation of CO_2_-equivalent emissions from the energy used by a LINAC to treat the 4 most commonly treated cancers in the United States with EBRT. In addition, we report the emissions associated with the daily operations of a LINAC. This information provides new insight into the components of cancer care that contribute to the carbon footprint of our health care system. The power consumption from LINACs while treatment is administered represents a small portion of CO_2_ emissions within the health care system. For comparison, previous reports of the carbon footprint of operating rooms ranged from 6 to 814 kg CO_2_ equivalents per surgery,^6^^,^^7^ whereas in the most common cancer types, ranges from 0.28 to 36.4 kg CO_2_ per course of EBRT in our report. It has also been described previously that the mean consumption per computed tomography (CT) scan was 1.2 kWh,^8^ and our study showed that courses of EBRT range from 1.83 to 85.68 kWh (median, 10.76; IQR, 6.41-22.55). Of note, it is worthwhile indicating that there may result in a fairly large range of emissions within a type of treatment (eg, conventional prostate) depending on the field size and inclusion of elective or regional nodal disease versus just the primary site.

There have been a few studies in Europe investigating energy efficiency for diagnostic radiology,^8^^,^^9^ as well as operating rooms,^10^ but this study presents the first to our knowledge that reports the carbon emissions of a LINAC during treatment with EBRT. Within this study, the “Standby” mode of the LINAC uses the most energy and provides the most significant magnitude of emissions. It has been reported previously that emissions associated with CT machine energy use are greatest in the idle state, which suggests that engineering redesign may yield considerable energy savings.^8^ Partnering with companies that are working in the space to engineer climate-conscious machinery for the diagnosis and treatment of cancer may be effective with offsetting CO_2_ equivalents and other GHG emissions within the health care sector.

Although our study aimed to only analyze the emissions of treatment alone, there are several limitations to our analysis that warrant future investigation. First, patient transportation and lodging were not included in our data. In a study using the UK Targeted Intraoperative Radiotherapy (TARGIT-A) trial comparing the carbon footprint of patient travel for single-dose intraoperative radiation therapy versus hypofractionated EBRT for breast cancer, the authors found that the carbon emissions were reduced to an average of 24.7 kg CO_2_ from 111 kg CO_2_.^11^ Future studies may be designed to capture exact driving times and distances per patient, as well as vehicle information to calculate the individual patient carbon footprint of EBRT. In addition, this study was performed assuming the highest power of the LINAC was used throughout the entirety of treatment. Generally, power used by the LINAC during beam-on time varies depending on the treatment characteristics (ie, the mechanical axes in operation and beam energy being used), and the current study may likely be presenting with overestimation.

To calculate the estimated CO_2_-equivalent emissions, we used the EPA calculator, a verified governmental, online resource as previously described. Although the EPA calculator is a useful tool to estimate the GHG equivalencies via CO_2_ equivalent emissions, there are several limitations that are worth noting for future studies. First, the EPA calculator uses a national average CO_2_-equivalent emissions factor (kg CO_2_/kWh), and would therefore either over- or underestimate the exact emissions based on the location of the LINAC within the country on the power grid and time of day differences in the power generation mix used to produce electricity. For example, the grid average in North Carolina is 0.308 kg CO_2_/kWh.^12^ In addition, the EPA calculator is an estimate of CO_2_ equivalents, which is generally used as a surrogate for GHG emissions and only includes CO_2_ as a GHG.

## Conclusion

Recent calls to action to physicians, especially to oncologists, have suggested strategies to minimize waste, advocate for partnership in more efficient machines and materials, and perform life-cycle analyses (LCAs) of radiation oncology supplies.^13^ Although our study did not aim to report complete carbon footprint of EBRT, we provide new information regarding the carbon emissions of the energy used by a LINAC during treatment. Furthermore, An LCA would be necessary to report the true carbon footprint. Future studies using an LCA method would capture the energy used for the clinic and treatment planning workflow would help give the most accurate estimate of emissions per course of treatment. This effort may include the use of metering of the treatment machine to report accurate energy usage. This would include any CT planning or simulation scans used for treatment planning, on-board imaging for daily setup and treatment verification, emissions of a working clinic including air conditioning and heating, and the construction of the machines (such as the LINAC). Given the clinical necessity of multiday treatments, strategies also may include assessment of the distribution of radiation centers in the United States, support for affordable housing in proximity to centers for those undergoing treatment, and support for mass transit infrastructure.

In addition, the GHG and CO_2_-equivalent emissions associated with electricity generation depends on location and time of day. The prevalence on the power grid of zero-carbon generation resources such as solar, wind, and nuclear varies by region of the United States, as does the extent to which lower-carbon fossil fuels like natural gas have replaced higher-emitting fuels such as coal. The mix of generation resources online also changes over the course of the day in a given region as electricity demand rises and falls. The emissions intensity (kg CO_2_/kWh) of the electricity used for treatment will therefore depend on the facility location and appointment time and differ from the national average we used here. A limitation to this study is that within our assumptions, the energy used was stable and does not account for the changes in source that would naturally occur throughout the day. To accurately record this information, one would need to have knowledge of the various power sources and consult with engineering and operations of the center, as well as the city or state government.

We have reported an estimate of CO_2_-equivalent emissions of a LINAC treating the 4 most common cancers treated with EBRT and identified limitations for future investigation and refinement of the estimate. It is evident that comprehensive studies, including LCAs, are warranted to minimize the environmental effects of health and cancer care.
